# Vitamin B5 and vitamin U review: justification of combined use for the treatment of mucosa-associated gastrointestinal pathologies

**DOI:** 10.3389/fphar.2025.1587627

**Published:** 2025-05-21

**Authors:** Valentin P. Shichkin

**Affiliations:** Aktipharm LLC, Kyiv, Ukraine

**Keywords:** vitamin B5, vitamin U, pantothenic acid, S-methylmethionine, gastrointestinal mucosa, inflammation, erosive lesions

## Abstract

Inflammatory and erosive lesions of the gastrointestinal mucosa encompass many conditions presenting significant challenges to public health worldwide. Amidst the quest for effective treatments, the attention of researchers has turned towards the potential therapeutic impact of bioactive nutrients, notably vitamin B5 (pantothenic acid) and vitamin U (S-methylmethionine). This review delves into the current understanding of the impact of these vitamins on the gastrointestinal system, analyzing their mechanisms of action and giving clinical evidence of the effectiveness of the combined application of these vitamins in the management of erosive gastrointestinal diseases.

## 1 Introduction

Inflammatory and erosive lesions of the gastrointestinal mucosa are common companions to a wide range of gastrointestinal pathologies, from *Helicobacter pylori* infection to drug-induced damage caused by the intake of nonsteroidal anti-inflammatory drugs, highlighting the global relevance of this issue for healthcare (Nguyen et al., 2022). This is facilitated by the fact that the gastrointestinal mucosa is continuously exposed to the harmful effects of so-called aggressive factors (hydrochloric acid, toxic substances, medications, microorganisms, food components, etc.), and nearly all modern standard therapies for such conditions are aimed at neutralizing the impact of these aggressive factors (Malfertheiner et al., 2022). However, despite the high effectiveness of standard treatment (anti-Helicobacter and antisecretory therapies), it does not ensure complete stabilization of the pathological process, and it has little effect on stimulating reparative processes in the mucosa, indicating the need for additional therapeutic intervention.

Indeed, the state of the mucosa is influenced not only by aggressive factors but also by protective factors (mucus, mucins, epithelial cell regeneration, blood circulation, etc.), the influence of which plays a significant role in both preventing and treating inflammatory and erosive lesions of the gastrointestinal mucosa (Pennelli et al., 2020; Cheng et al., 2021). This is especially relevant for elderly patients due to additional conditions that reduce the activity of protective factors, specifically age-related decreases in the production of components of the mucus-bicarbonate barrier and reduced microcirculation due to chronic diseases such as atherosclerosis, diabetes, and others (Koffeman et al., 2014; Cristina and Lucia, 2021). This often occurs against the backdrop of long-time medication use (primarily aspirin and nonsteroidal anti-inflammatory drugs) which increases the negative impact on the mucosa of the upper gastrointestinal tract, promoting the development of inflammatory and erosive processes. Therefore, in addition to the standard suppression of aggressive factors, it is now considered beneficial to additionally target protective factors, as there are frequent cases when even successful standard therapy does not fully eliminate the pathological process and requires additional treatment. A notable example is the persistence of inflammation in the gastric mucosa, according to various authors, in 28.2%–58.6% of patients even 1 year after successful eradication of *Helicobacter* (Osaki et al., 2008; Tulassay and Herszenyi, 2010).

It is known that the gastrointestinal mucosa is lined with epithelial tissue, the integrity and functioning of which are associated with gut microbiota and disrupted in inflammation and erosive diseases (Okamoto and Watanabe, 2016; Okumura and Takeda, 2017; Kong et al., 2018; Huang et al., 2024). The long persistence of inflammatory processes in the mucosa is equivalent to a continued high risk of its further damage and malignancy, necessitating the inclusion of components in the treatment regimen that protect the mucosa from lesions and support its regeneration. This focus on the reparative effect is because inflammatory and erosive lesions of the gastrointestinal mucosa are characterized by delayed regeneration against the background of degenerative changes in the epithelial layer, which requires an additional correction (Guan, 2019; Holtmann et al., 2024; Papaiakovou et al., 2024). As known, the restoration of the epithelium occurs through the proliferation and differentiation of stem cells, which are sensitive to the effects of certain vitamins (Fedirko et al., 2009; Godoy-Parejo et al., 2020; Li et al., 2023; Sirajudeen et al., 2023). In this regard, the potential therapeutic role of such bioactive nutrients as vitamin B5 (pantothenic acid, dexpanthenol) and vitamin U (S-methylmethionine, methylmethionine sulfonium chloride), which have pronounced reparative properties concerning the epithelium and which stand out as promising candidates for additional or alternative therapy of inflammatory and erosive gastrointestinal pathologies is high attractive.

The protective and restoring effects of vitamin U in erosive disorders of the gastrointestinal mucosa are well-documented (Patel and Prajapati, 2012; Ivashkin et al., 2020; Drozdov et al., 2023). Some data also suggest similar effects of vitamin B5 on the integrity of the intestine epithelial-mucosal barrier (Wang et al., 2024). Moreover, clinical research indicates that a combination of vitamin B5 and vitamin U in therapeutic doses is effective in healing erosions of the gastrointestinal mucosa with a minimum of side effects (Gubergrits et al., 2013; Gubergrits et al., 2014; Danylchenko and Tokarenko, 2014; Bondarenko et al., 2015; Kolesnikova and Solomentseva, 2017; Babinets et al., 2019; Babinets and Machnitska, 2019; Babinets and Machnitska, 2021). This result is achieved through a mutually potentiating combination of vitamin B5 and vitamin U effects, which have regenerative and protective impacts on the epithelial tissue of the gastrointestinal tract.

This review briefly summarizes current knowledge concerning vitamin B5 and vitamin U and analyzes mechanisms through which these vitamins may impact the regeneration of the gastrointestinal mucosa. A special focus on the justification of using a combination of vitamin B5 and vitamin U, and the clinical outcomes using this combination for the mucosa erosive damage of the upper gastrointestinal tract.

## 2 Vitamin B5 (pantothenic acid)

### 2.1 Sources of vitamin B5

Vitamin B5, also known as pantothenic acid, is a water-soluble vitamin. It serves as the precursor and a crucial component of coenzyme A (CoA) playing a pivotal role in various metabolic and signaling pathways essential for cellular functions such as cell growth, intermediary metabolism, and neurotransmitter synthesis (Dibble et al., 2022; Hrubša et al., 2022; Sanvictores and Chauhan, 2025).

The biosynthesis of vitamin B5 occurs in plants, fungi, and most bacteria. Humans and animals cannot synthesize this vitamin and depend on its exogenous supply from food. Therefore, the homeostasis of vitamin B5 in the human body is assured mainly by a food diet. An additional possible source is gut microbiota metabolism. Furthermore, the degradation of endogenous CoA may also impact the level of vitamin B5 in the body (Ottenhof et al., 2004; Webb et al., 2004; Leonardi and Jackowski, 2007; Roje, 2007; Martinez et al., 2014; Magnusdottir et al., 2015; Meir and Osherov, 2018; Yoshii et al., 2019; Hrubša et al., 2022).

Vitamin B5 is widely distributed in foods of both plant and animal origin. The body does not store vitamin B5 and, therefore, it needs to be replaced daily with food (Hanna et al., 2022; Hrubša et al., 2022). Major sources include meat, liver, kidney, eggs, milk, cheese, fish, nuts, mushrooms, yeast, whole grain cereals, legumes, broccoli and cauliflower, avocado, potatoes, sunflower seeds, lentils, and tomatoes. Small amounts of pantothenic acid are typically found in nearly all food (Hanna et al., 2022; Hrubša et al., 2022). However, about 85% of pantothenic acid found in food comprises CoA and phosphopantetheine (Ross et al., 2020; Munteanu and Schwartz, 2024). Additionally, pantothenic acid (as calcium pantothenate, sodium pantothenate, or dexpanthenol) is added to various foods to prevent deficiency due to incorrect nutrition or malnutrition or for certain nutritional requirements such as baby foods, athletes’ products, low-calorie, reduced-calorie, and vitamin-rich foods (Agostoni et al., 2014; Hrubša et al., 2022).

In humans and animals, the source of vitamin B5 is also the intestinal microbiota. Nearly all *Bacteroides* and Proteobacteria can produce pantothenic acid and CoA (Magnusdottir et al., 2015). The majority of bacteria including *Escherichia coli*, *Salmonella typhimurium*, and *Corynebacterium glutamicum* produce pantothenate from aspartate as an intermediate product in the biosynthesis of valine (Begley et al., 2001; Leonardi and Jackowski, 2007; Martinez et al., 2014; Pietrocola et al., 2015; Bourgin et al., 2022; Hossain et al., 2022; Wan et al., 2022). However, the extent of this contribution is unknown (Kelly, 2011; Said, 2011; Yoshii et al., 2019). At the same time, little is known also about the impact of vitamin B5 supplements on gut microbiota. Different effects of pantothenic acid on the abundance and diversity of intestinal microbiota indicated that vitamin B5 might change the intestinal microbiota growth profile and biological function (Carrothers et al., 2015; Hossain et al., 2022; Wan et al., 2022; Wibowo and Pramadhani, 2024) and thus, possibly, affect the immune system in a sense its mobilization for protection from food allergies and cancer (Shichkin et al., 2023; Shichkin, 2024).

### 2.2 Role of vitamin B5 in cell metabolism and ATP synthesis

Vitamin B5 participates in many biochemical and signaling reactions. In particular, it is essential for CoA and acyl-carrier protein (ACP) synthesis in yeast and mammalian cells (Martinez et al., 2014; Rucker, 2016; Gheita et al., 2020; Hanna et al., 2022). ACP and CoA are necessary to synthesize fatty acids, cholesterol, acetylcholine, and bile acids and they are vital for many other biochemical reactions (Leonardi and Jackowski, 2007; Manolopoulos et al., 2008; Davaapil et al., 2014; Parthasarathy et al., 2019). Primary, ACP is expressed in the inactive form. For its activation, the attachment of a prosthetic group during the reaction with CoA catalyzed by 4′-phosphopantetheinyl transferase is required. CoA is a thiol that activates the carboxylic groups in the thiol esters forming process. As such, it is involved in several key metabolic pathways in the Krebs cycle including fatty acid biosynthesis and oxidation (Pietrocola et al., 2015; Sibon and Strauss, 2016). CoA is required for processing large organic molecules, such as lipids, carbohydrates, and proteins (Yoshii et al., 2019; Hrubša et al., 2022). These reactions generate energy with the forming of acylated forms of CoA, such as acetyl-CoA, succinyl-CoA, propionyl-CoA, isovaleryl-CoA, isobutyryl-CoA, αmethylbutyryl-CoA, and fatty acyl-CoA (Slyshenkov et al., 2004; Czumaj et al., 2020). Therefore, pantothenic acid being a substrate for CoA is involved in ATP synthesis and increasing mitochondrial activity due to the role of CoA in fat and carbohydrate metabolism, and less via protein metabolism (Slyshenkov et al., 2004; Gheita et al., 2020). Food-derived vitamin B5 in this process is converted to pantethine and then to acetyl-CoA and ACP (Wan et al., 2022). These two compounds are critical in burning fats and carbohydrates in energy metabolism (Slyshenkov et al., 2004; Webb et al., 2004).

Participating in CoA and acetyl-CoA synthesis vitamin B5 is used as a mechanism for moving carbon atoms inside cells promoting carbons to enter the Krebs cycle during glycolysis and conversion of α-ketoglutarate to succinate. Glucose and glutamine are the two main carbon sources contributing to the Krebs cycle (Pavlova and Thompson, 2016; Still and Yuneva, 2017). Pantothenic acid plays a crucial role in the conversion of pyruvate to acetyl-CoA and α ketoglutarate to succinyl-CoA both of which are required for the stimulation of the tricarboxylic acid cycle in the energy-producing lipid metabolism resulting in the synthesis of ATP molecules (Parthasarathy et al., 2019; Hrubša et al., 2022). However, the ATP supply is limited and constantly replenished through phosphorylation processes during respiration, fermentation, and photosynthesis. ATP is renewed extremely quickly. In humans, the lifespan of one ATP molecule is less than 1 minute and the human body synthesizes about 40 kg of new ATP molecules a day. Therefore, pantothenic acid as a promoter for ATP synthesis increases the reparative processes, and this property of vitamin B is used in drugs evoking the skin epithelium and mucous membranes at damage (Kehrl and Sonnemann, 2000; Kehrl et al., 2003; Wananukul et al., 2006; Wollina and Kubicki, 2006; Udompataikul and Limpa-o-vart, 2012; Shanazi et al., 2015; Heise et al., 2019; Hrubša et al., 2022).

Via CoA signaling pathways vitamin B5 regulates the synthesis of different steroid hormones and some chemical mediators (Slyshenkov et al., 2004). In particular, acetyl-CoA is required to produce steroid adrenal hormones including cortisol (Wan et al., 2022). Also, the metabolic pathways in white blood cells actively convert vitamin B5 into coenzyme CoA. The high intake of B5 increases the activity of CoA, which can lead to an exacerbated production of immunologically active compounds that play a role in the body’s defenses, such as acute response proteins associated with inflammation, pro-inflammatory cytokines, and adhesion molecules (Lee et al., 2018). The interruption of these pathways can lead to unexpected inflammatory reactions. The pantothenic acid metabolism, among others, affects oxidative stress, cellular adhesion, and polynuclear cell effectiveness (Nitto and Onodera, 2013).

### 2.3 Pharmacokinetics of vitamin B5

#### 2.3.1 Intestinal metabolism

Cells uptake pantothenic acid via MYC which is recognized as a master regulator of metabolism inducing glycolytic flux and increasing glutaminolysis (Dong et al., 2020). MYC enhances pantothenic acid uptake in a cell-autonomous manner (Kreuzaler et al., 2023). Pantothenic acid is transported into cells alongside biotin (vitamin B7) and ɑ-lipoic acid via the sodium-dependent multivitamin transporter (SMVT) also known as SLC5A6 (Prasad et al., 1998). Vitamin B5 can be absorbed only in the small intestine (Halsted, 2003). Intestinal deletion of SMVT results in stunted growth, spontaneous and severe inflammation, increased gut permeability, and early death (Sabui et al., 2018). Finding that the SMVT facilitates the transport of both vitamin B5 and vitamin B7 suggests that these vitamins play a role in maintaining gut homeostasis (Peterson et al., 2020).

**FIGURE 1:**
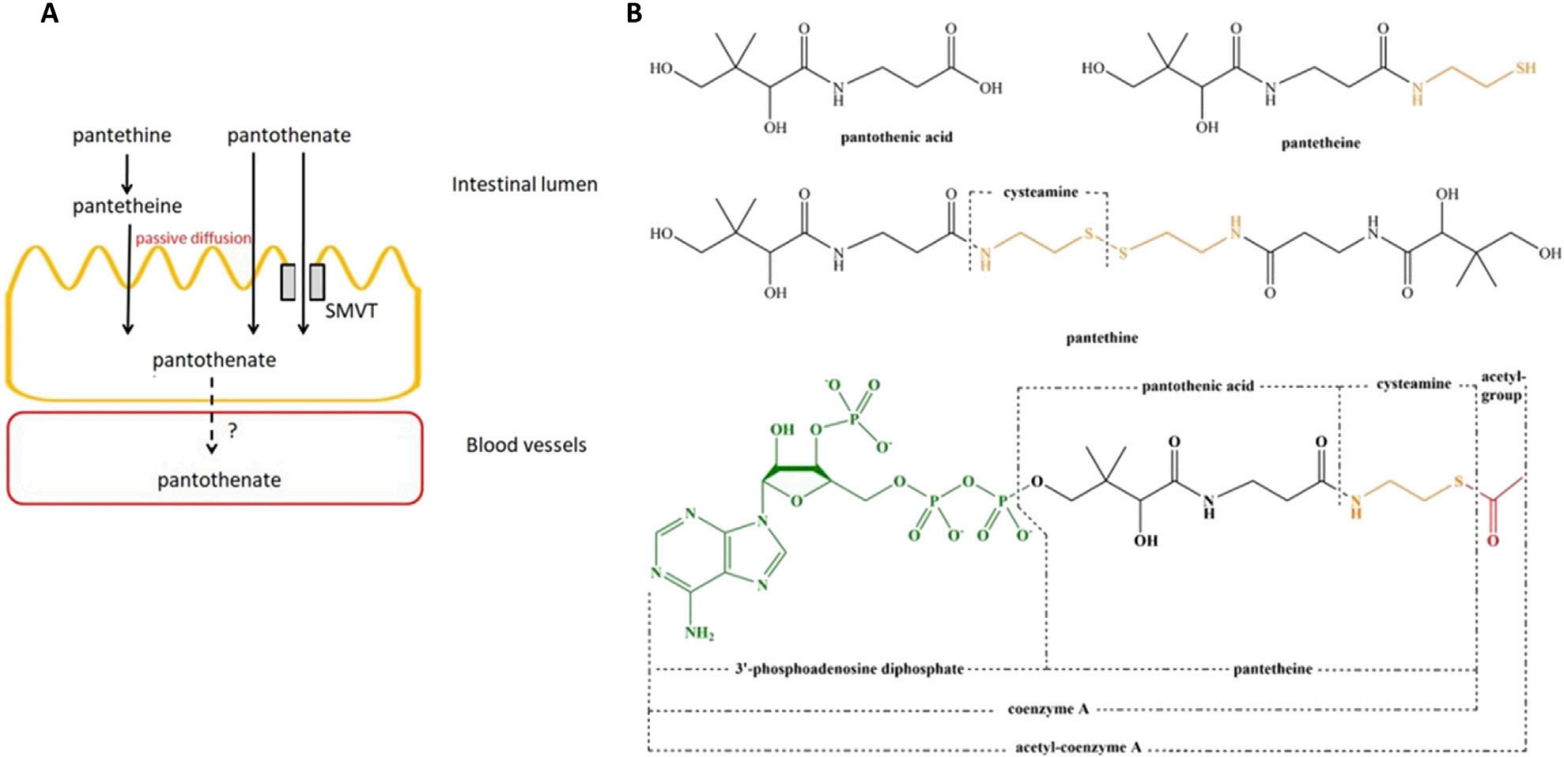
Membrane transport and structure of vitamin B5 and its derivates. **(A)**, Membrane transport of vitamin B5 and its derivates in the small intestine. **(B)**, structure of pantothenic acid, pantetheine, pantethine, and acetyl-CoA. Abbreviation: SMVT, sodium-dependent multivitamin transporter. Adopted from Hrubša et al. (2022). This open access article is distributed under the terms and conditions of the Creative Commons Attribution (CC BY) license (https://creativecommons.org/licenses/by/4.0/).

The intestinal tract is exposed to two sources of vitamin B5, diet and bacteria origin (Wan et al., 2022). Dietary vitamin B5 exists mainly in the form of CoA which is hydrolyzed to pantetheine by alkaline phosphatase and then quickly converted into the absorbable forms in the intestinal lumen with the help of pantetheinase enzyme (vanin) (Czumaj et al., 2020; Naquet et al., 2020; Wan et al., 2022). As a result, pantothenate and cysteamine are generated (Nitto and Onodera, 2013). Similarly, pantethine (disulfide derivative of pantothenic acid and a stable form of pantetheine) is hydrolyzed to a considerable extent (80%) to pantetheine and further to pantothenate at passing the intestinal wall (Hrubša et al., 2022). At low concentrations in the intestinal lumen, pantothenic acid is actively transported via SMVT. At high concentrations of vitamin B5, passive diffusion occurs. There is no significant difference in the transport rate of vitamin B5 derivates in different segments of the intestine (Shibata et al., 1983; Said et al., 1998; Prasad et al., 1999; Hrubša et al., 2022) (Figure 1A). It is indicated that absorption of the bacterially synthesized vitamin B5 in the large intestine also involves the SMVT system, though direct evidence is lacking (Ghosal et al., 2013; Wan et al., 2022). In the colon, vitamin B5 is absorbed with the help of the same transporter SMVT similar to the small intestine (Said et al., 1998). The structure of pantothenic acid and its derivatives is shown in Figure 1B.

#### 2.3.2 Metabolism in blood and tissues

There is no data regarding the mechanism of pantothenic acid transport from the enteric cells via the intestinal basolateral membrane into the bloodstream. In blood, pantothenate is transported in the free form not binding to albumin (Karnitz et al., 1984). Tissues and red blood cells rapidly take up pantothenic acid from the blood. Its levels in red blood cells are higher than in plasma (Eissenstat et al., 1986). The transport into red blood cells is mediated by passive diffusion. Erythrocytes have a limited metabolizing ability to pantothenate, and they are used probably only as a pantothenate transporter to other tissues. In red blood cells, pantothenic acid could be converted to 4′-phosphopantothenic acid, but not to CoA (Annous and Song, 1995). Therefore, with the red blood cells, pantothenic acid is transported as pantothenate, 4′-phosphopantothenic acid, and pantetheine (Shibata et al., 1983; Annous and Song, 1995). The uptake of these metabolites into different tissues such as the liver, heart, kidney, lungs, adipose tissue, and placenta occurs via SMVT (Vadlapudi et al., 2012). This transporter also enables the transport through the blood-brain barrier. In addition, the distribution studies in rats confirmed that vitamin B5 is accumulated in tissues, and only a low concentration remains in the blood (Shibata et al., 1983; Hrubša et al., 2022).

#### 2.3.3 Excretion

Pantothenic acid is mainly excreted via urine. The degree of urinary excretion of vitamin B5 depends on the amount of the vitamin in the diet. In pharmacokinetics studies its excretion was relatively high ranging from 60% to 72% suggesting that its content in the diet is mostly sufficient (Eissenstat et al., 1986; Tsuji et al., 2010; Hrubša et al., 2022).

#### 2.3.4 Stability

Pure pantothenic acid is a viscous liquid. Vitamine B5 is more stable in a slightly alkaline environment. Maximum stability appears at pH 5–7. Its degradation is accelerated by heat. It is moderately stable to atmospheric oxygen and light when protected from moisture. Vitamin B5 is more sensitive to heating than light and oxygen (Nurit et al., 2016). Pantothenic acid is quite stable during thermal processing at pH 5–7 and food cooking but food processing may alter the content of pantothenic acid (Ottaway, 2010; Godoy et al., 2021; Hrubša et al., 2022).

Pantothenic acid is generally administered in solid form as calcium pantothenate. This solid form is more stable against light, heat, and oxygen than pantothenic acid but unstable in alkaline and acidic conditions. Another solid form of pantothenic acid is sodium pantothenate. Its use, however, is limited due to high hygroscopicity (Schnellbaecher et al., 2019; Godoy et al., 2021). A synthetic derivative of pantothenic acid dexpanthenol (D-panthenol) is used for therapeutic purposes. Dexpanthenol is provitamin B5. In its molecule, the acid group is replaced by an alcohol group (Shin et al., 2021). In animal and human bodies, dexpanthenol is easily converted into pantothenic acid and they have similar vitamin activity.

### 2.4 Therapeutic and health impact of vitamin B5

Dexpanthenol is mainly used for topical treatment of skin and mucous lesions. Its effects are mediated by the moisturizing effect based on hygroscopic properties. Dexpanthenol improves skin hydration and supports skin recolonization with commensal bacteria (Stettler et al., 2017). As believed, the dexpanthenol moisturizing effect is also linked to the ability to regenerate the epidermal barrier through increased epithelial differentiation and lipid synthesis. It protects epithelium from apoptosis and probably promotes cellular proliferation (Shin et al., 2021; Szumny and Misiuk-Hojło, 2022). Therefore, it helps to recover the epidermal barrier function and has anti-inflammatory activity supporting more fust wound closure (Kehrl and Sonnemann, 2000; Kehrl et al., 2003; Wananukul et al., 2006; Wollina and Kubicki, 2006; Udompataikul and Limpa-o-vart, 2012; Shanazi et al., 2015; Heise et al., 2019).

These properties of vitamin B5 may contribute to the repair of mucosal intestinal lesions and support gut microbiota homeostasis. Thus, one study explored the effects of different vitamin B5 doses on the intestinal growth and function in piglets. The addition of 50 mg/kg vitamin B5 enhanced the morphological structure, cell proliferation, and differentiation in the ileum, cecum, and colon. This study also reported a significant decrease in the relative abundance of harmful bacteria and a significant increase in beneficial bacteria in the intestine (Wang et al., 2024). The positive effect of vitamin B5 on the balance of intestinal microbiota has also been noted in several other original and review papers (Hossain et al., 2022; Wan et al., 2022; Wibowo and Pramadhani, 2024). This is especially important in populations of aging people with impaired digestive function (Cristina and Lucia, 2021), for whom the restoration and maintenance of beneficial microflora is a critical factor in maintaining a high quality of life.

A recent study reported that vitamin B5 and CoA favor the cytotoxic CD8^+^ T-cell differentiation into interleukin-22 (IL-22)-producing Tc22 cells likely through fueling mitochondrial metabolism. It is considered, that Tc22 cells are the cells with the strongest antitumor activity among CD8^+^ T-cell subsets. According to current literature, the increased circulating levels of vitamin B5 should be correlated with a good state of health reflected in effective immune responses (Paul et al., 2021; Bourgin et al., 2022; Hong et al., 2022). The plasma level of vitamin B5 positively correlates with response to PD-1-targeted immunotherapy in melanoma patients. Supplementation with vitamin B5 increases the efficacy of PD-L1-targeted cancer immunotherapy in mice, and the culture of T cells with CoA *in vitro* enhances T-cell antitumor activity upon adoptive transfer to mice. These findings suggest vitamin B5 might stimulate anticancer immunosurveillance (Bourgin et al., 2022). Additionally, B5 derivatives decrease tumor necrosis factor-alpha (TNF-α) levels and reduce oxidative stress in patients with endometriosis (Karapinar et al., 2017).

Oxidative stress plays an essential role in the pathogenesis of various gastrointestinal diseases, including peptic ulcers, gastrointestinal cancers, and inflammatory bowel disease. Reactive oxygen species (ROS) are generated as by-products of normal cellular metabolic activities within the gastrointestinal tract. Despite the protective barrier provided by the mucosa, food, drugs, and microbial pathogens can induce oxidative injury and gastrointestinal inflammatory responses involving the epithelium and inflammatory immune cells (Bhattacharyya et al., 2014). High vitamin B supplementation could alleviate lipid content accumulation and oxidative damage associated with fat oxidation (Qian et al., 2022). Therefore, due to its antioxidant activity, vitamin B5 may directly contribute to the gastrointestinal protection from oxidative damage. Since vitamin B5 is incorporated into coenzyme A, it protects cells against peroxidative damage by increasing the level of glutathione (DrugBank, 2025).

Alongside other vitamins, vitamin B5 may improve mental health (Mikkelsen and Apostolopoulos, 2019), and cells cultured in the presence of vitamin B5 have an improved metabolic capacity compared to unexposed cells (Pourcel et al., 2020). Physiological-acceptable levels of vitamin B5 are effective in wound healing, liver detoxification, and joint health support (Erdmann et al., 2021).

The therapeutic dose of pantothenic acid or dexpanthenol is 10–50 mg/day when administered intramuscularly or up to 400–800 mg/day at oral administration (Gaddi et al., 1984; Arsenio et al., 1984; Donati et al., 1989; Coronel et al., 1991; Evans et al., 2014; Hrubša et al., 2022). Adults should take 5 mg/day, pregnant women 6 mg/day, and lactating women 7 mg/day. For oral administration, it is recommended to take with food (Sanvictores and Chauhan, 2025). In clinical studies, the doses of pantethine reached 600 or 900 mg/day and were administered to patients suffering from different forms of dyslipidemia and diabetes mellitus (Gaddi et al., 1984; Arsenio et al., 1984; Donati et al., 1989; Evans et al., 2014; Hrubša et al., 2022). Dexpanthenol is also used topically in 2%–5% concentration as a cream, emollient, drops, gel, lotion, oil, ointment, solution, and spray on skin injuries and mucous lesions (Proksch et al., 2017).

#### 2.4.1 Vitamin B5 deficiency

Vitamin B5 deficiency is generally rare since it is submitted in various foods but its deficiency could be observed in people and animals with severe malnutrition. The most common symptoms of pantothenic acid deficiency in animals are growth problems, skin rash, gastrointestinal disorders, and nervous symptoms, such as ataxia, loss of coordination, and muscle weakness. Human studies also showed similar symptoms (Hrubša et al., 2022). Among the reported abnormalities associated with vitamin B5 deficiency are cerebral and eye defects, digital hemorrhages and edema, interventricular septal defects, anomalies of the aortic arch pattern, hydronephrosis, clubfoot, cleft palate, increased arthritis pain, fatigue, irritability, headaches, gastrointestinal issues, the sensation of weakness, and dermal defects (Gheita et al., 2020; Hanna et al., 2022; Hrubša et al., 2022; Wan et al., 2022). Pantothenic acid deficiency impact on impaired adrenal cortical function. Lower levels of blood pantothenic acid were observed in patients with rheumatoid arthritis, and the severity of arthritis negatively correlated to the vitamin B5 level (Wan et al., 2022). Low serum B5 levels are also associated with an increased incidence of hypertension. Nearly all symptoms resolve after resuming intake of pantothenic acid (Gheita et al., 2020; Hanna et al., 2022; Wan et al., 2022).

The pantothenic acid deficiency is possible in people with mutations in the pantothenate kinase 2 (PANK2) gene. The PANK2 gene mutations decrease PANK2 ferment activity which might diminish the conversion of vitamin B5 to CoA and result in its lower levels. The mutation of PANK2 leads to pantothenate kinase-associated neurodegeneration and increases the risk of Parkinson’s disease and other oxidative stress disorders (Johnson et al., 2004; Hrubša et al., 2022). Levels of pantothenic acid excretion below 1 mg/day usually indicate deficiency. Serum pantothenate concentration does not correlate with its status but a level of less than 50 mcg/mL can be considered a deficiency diagnosis (Hanna et al., 2022).

#### 2.4.2 Toxicity

Vitamin B5 has a very low acute toxicity. In a set of research, no toxicity symptoms were observed at oral administration of 1 g/kg of calcium pantothenate to dogs and monkeys, and any pathological changes in examined organs were also absent. The LD50 for mice and rats was 10 g/kg at the oral administration. Respiratory failure occurred over 2 hours following i. v. and i. p. administration and over 6–12 h after oral and s. c. administration of the lethal dose (Hrubša et al., 2022).

Similarly, chronic toxicity of vitamin B5 is very low. Hence, this vitamin is generall considered safe (Hrubša et al., 2022). No symptoms of chronic toxicity nor pathological organ changes were detected in rats fed 50 or 200 mg/daily of calcium pantothenate during 190 days nor in dogs (daily dose 50 mg/kg) and in monkeys (1 g daily for 4–5 kg body weight) fed for 6 months. No tolerable upper intake limit for pantothenic acid has been established for humans. Massive doses of calcium pantothenate (10–20 g/day) can cause mild diarrhea. Pantethine is also well tolerated and in therapeutic doses has no side effects (Arsenio et al., 1984). Few patients treated with high doses of pantethine (900 mg/day) complained of mild gastric discomfort and pruritus (Donati et al., 1989; Coronel et al., 1991; Hrubša et al., 2022).

#### 2.4.3 Adverse effects and precautions

The effects of vitamin B5 depend primarily on the dose. Large doses might cause diarrhea and functional disorders of the digestive tract (Chawla and Kvarnberg, 2014). Consumption of vitamin B5 might correlate with cognitive disorders (Lee et al., 2018). It was observed that baseline levels of blood plasma vitamin B5 are associated with an increased risk of death in patients with hypertension, particularly in older adults and those who have high folate levels (Hong et al., 2022; Munteanu and Schwartz, 2024). The potential negative impact of vitamin B5 high doses may include semicoma, Reye-like syndrome, and encephalopathy (Noda et al., 1988), gastrointestinal disorders, and liver dysfunctions in the elderly (Erdmann et al., 2021; Chazot et al., 2022), and higher rates of genome damage as a biomarker for higher risk of cancer (Fenech et al., 2005; Munteanu and Schwartz, 2024). Vitamin B5 intake might correlate with increased cerebral amyloid-beta peptide burden in individuals with cognitive impairment. Some research suggests that cognitive impairment is a potential contraindication for vitamin B5 intake (Lee et al., 2018). Patients with an allergy to pantothenic acid or its ingredients should also not take vitamin B5 (Sanvictores and Chauhan, 2025).

#### 2.4.4 Drug interaction

At least 63 drugs, as reported, have interactions with vitamin B5 (DrugBank, 2025). From these, azithromycin, clarithromycin, erythromycin base, erythromycin ethyl succinate, erythromycin lactobionate, erythromycin stearate, and roxithromycin have moderate interactions with vitamin B5. Other drugs have mild interactions with vitamin B5 (Sanvictores and Chauhan, 2025). Most interacting drugs decrease the effect of vitamin B5. While such drugs as alpha-1-proteinase inhibitor, aprotinin, hydrogen peroxide, menadione, and tranexamic acid may increase the thrombogenic activities of vitamin B5. The risk or severity of hypercoagulability can be increased when vitamin B5 is combined with fitusiran. The risk or severity of adverse effects can also be increased when aminocaproic acid is combined with pantothenic acid (DrugBank, 2025). The interaction of vitamin B5 supplements with other nutrients is unstudied (Hanna et al., 2022).

Summarizing above, we can highlight that the vitamin B5 protective and regenerative effects concerning gastrointestinal tract may be exerted through direct mechanisms, which include supporting beneficial gut microbiota balance, increasing antioxidant defenses, and impacting gut epithelial barrier integrity, vie mitigating inflammation-associated damage within the gastrointestinal tract, and through indirect mechanisms mediated its participation in cellular metabolism resulting in ATP energy synthesis. The immunomodulatory properties of vitamin B5 can probably also contribute to its gastroprotective effect. However, while there are single optimistic experimental studies on animal models, no direct clinical data confirm the gastroprotective effect of vitamin B5 in humans.

## 3 Vitamin U (S-methylmethionine)

### 3.1 Sources of vitamin U

S-methylmethionine sulfonium chloride (S-methylmethionine, methylmethionine, vitamin U) is a vitamin-like compound. This is an activated derivative of the essential amino acid methionine. S-methylmethionine is not synthesized in the human body and must come from outside including with foods containing it (white cabbage and its juice, asparagus, broccoli, fresh potato juice, carrots, celery, beets, parsley and dill, green onion feathers, tomatoes, bananas, green tea, raw egg yolks, raw milk, liver, etc.). S-methylmethionine was originally identified in cabbage juice in 1952 by Cheney and called vitamin U (from the Latin ulcus – ulcer; anti-ulcer vitamin) because it inhibited ulceration in the digestive system and its impressive effects on gastrointestinal ulcer treatment (Cheney, 1950; 1952). Cheney’s research laid the foundation for using vitamin U to treat gastroenteric peptic ulcer diseases.

Vitamin U actively affects the secretory function of the stomach and mucous reparation. The daily intake of methylmethionine depends on age and gender. The recommended dose is about 200 mg daily (Kruchinina et al., 2018; Topchiy and Toporkov, 2019).

### 3.2 Vitamin U metabolism

S-methylmethionine is necessary for synthesizing all protein compounds in the human body. This is a functional hydrophilic molecule having a cationic structure with an α-amino acid end group (Patel and Prajapati, 2012). Due to a functional sulfonium group, S-methylmethionine is an intermediate link in many metabolic pathways in the human body.

**FIGURE 2:**
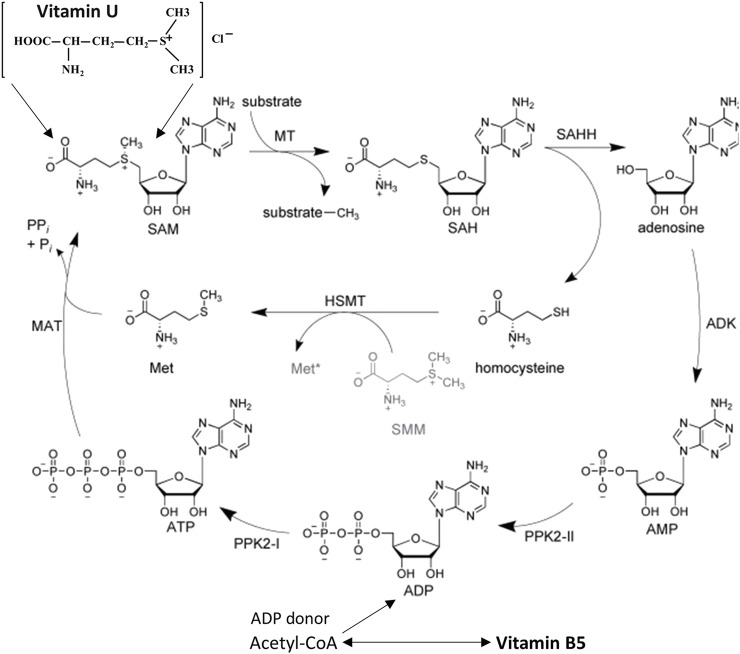
Role of vitamin U and vitamin B5 in the S-adenosyl-L-methionine regeneration cycle using enzyme-substrate reactions. Abbreviation (enzymes): MAT, L-methionine adenosyltransferase; MT, methyltransferase; SAHH, S-adenosyl-L-homocysteine hydrolase; ADK, adenosine kinase; PPK, polyphosphate kinase; HSMT, L-homocysteine S-methyltransferase. Abbreviation (substrates): ATP/ADP/AMP, adenosine 5′-tri/di/monophosphate; Met, L-methionine; SMM, S-methyl-L-methionine (vitamin U); SAM, S-adenosyl-L-methionine; SAH, S-adenosyl-L-homocysteine. Modified from Abdelraheem et al. (2022). This open access article is distributed under the terms and conditions of the Creative Commons Attribution (CC BY) license (https://creativecommons.org/licenses/by/4.0/).

The multi-stage biosynthesis of S-methylmethionine occurs through the conversion of L-methionine to S-adenosylmethionine (SAM) followed by the replacement of the adenosyl group on a methyl group with the participation of methionine-S-methyltransferase enzyme (Patel and Prajapati, 2012; Kruchinina et al., 2018) (Figure 2). S-methylmethionine is an activated form of methionine. It might participate in all methylation reactions using SAM as another activated form of methionine. SAM is an inhibitor of the most important enzyme of the methylation system which completes the formation of methyl radicals from one-carbon compounds restoring the methylene group of folates to methyl. Since SAM has an inhibitory effect on the system of the xenobiotic metabolism in the liver while S-methylmethionine does not affect the functional activity of the liver’s isoenzymes and it does not have an inhibitory effect on methylation processes, the use of the last one is preferable and safe as a nutrient (Kruchinina et al., 2018). Biotransformation of vitamin U activity proceeds in the liver, kidneys, and digestive tract. Metabolism of vitamin U in the liver and kidneys was found to proceed via methylation of homocysteine with the formation of methionine and via enzymatic hydrolysis to dimethylsulfide and homoserine. In the digestive tract, only the activity of S-methylmethionine sulphonium hydrolase was found (Patel and Prajapati, 2012).

SAM, the active form of methionine, is formed from ATP and methionine using the methionine adenosyltransferase enzyme (Abdelraheem et al., 2022). Due to the functions of pantothenic acid, a sufficient amount of ATP accumulates in cells, and SAM can be synthesized more actively than with ATP deficiency. SAM is a coenzyme that participates in the transfer reactions of methyl groups (Patel and Prajapati, 2012; Abdelraheem et al., 2022). It is requested in such cellular metabolic pathways as transmethylation, transsulfuration, and aminopropylation. The methyl group (CH3) attached to the sulfur atom of the methionine molecule in SAM is chemically active. Therefore, a methyl group can be transferred to a substrate molecule in a transmethylation reaction. More than forty metabolic reactions require the transfer of a methyl group from SAM to substrates such as nucleic acids, proteins, and lipids. This reaction is catalyzed by the methionine adenosyltransferase enzyme, which is present in all cell types. The structure (-S+-CH3) in SAM is an unstable group which determines the high activity of the methyl group (hence the term “active methionine”). This reaction is unique in biological systems because it appears to be the only known reaction that actively utilizes ATP and releases all three of its phosphate residues (Abdelraheem et al., 2022) (Figure 2).

### 3.3 Mechanisms of vitamin U therapeutic effect

**FIGURE 3:**
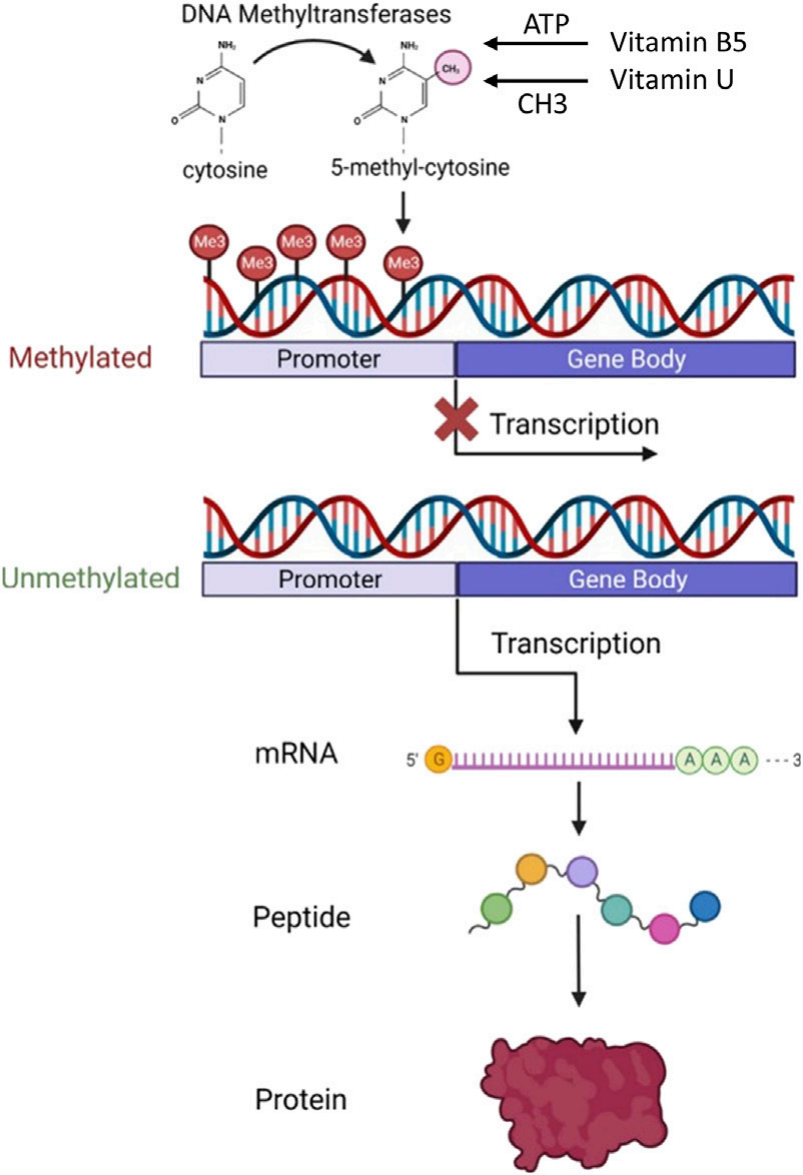
Role of vitamin B5 and vitamin U in DNA methylation and protein synthesis. The diagram shows how DNA methylation at the gene’s promoter region affects transcription and protein expression levels. Abbreviation: ATP, adenosine 5′-triphosphate. Modified from Wang et al. (2023). This open access article is distributed under the terms and conditions of the Creative Commons Attribution (CC BY) license (https://creativecommons.org/licenses/by/4.0/).

The main therapeutic effect of vitamin U is because S-methylmethionine is an active donor of methyl groups necessary for the DNA, RNA, and protein methylation which regulates DNA replication and protein synthesis resulting in cell division (Patel and Prajapati, 2012; Abdelraheem et al., 2022; Liu et al., 2023) (Figure 3).

Methyl is a chemical compound that is vital to the cell life cycle. Methyl groups are the building blocks for the most important protein molecules and DNA. The health of the cell and its lifespan ultimately depend on the DNA state. DNA methylation is responsible for the genome reorganization into silent and active regions of transcription. Therefore, methyl groups are a necessary trigger compound for DNA activation, renewal of essential proteins, and cell rejuvenation. Cells can correctly renew and function only with a sufficient amount of methyl groups (Abdelraheem et al., 2022; Liu et al., 2023).

Recent *in vivo* and *in vitro* evidence has revealed that various alterations of DNA methylation within intestine epithelial cells and smooth muscle cells may result in diverse cellular phenotypes including modified differentiation, apoptosis, and uncontrolled growth. Aberrant DNA methylation patterns are often found in cells with abnormal growth and differentiation including cells involved in intestinal pathologies such as inflammatory bowel diseases and intestinal pseudo-obstructions indicating a crucial connection between DNA methylation and cell development in the gastrointestinal system and their response to environmental signals (Jorgensen and Ro, 2019; Lorzadeh et al., 2021; Liu et al., 2023).

Several recent experimental studies also assume other mechanisms of the vitamin U protective effect that can be potentially realized in the gastrointestinal tract.

In the experimental study on rats with induced liver cancer, vitamin U exerted a significant antitumor effect mediated by a decrease in lipid peroxide formation, the expression level of inflammatory (TNF-α) and immunoregulatory (TGF-1β) cytokines, and the induction of nitric oxide synthase and glypican 3 (Abouzed et al., 2021).

The experimental study on mice have shown the positive regulation of *Sult1e1*, *Phlda1*, and *Ciart* genes in the liver with vitamin U food supplementation, suggesting that vitamin U may regulate xenobiotic, glucose, and circadian rhythm pathways (Egea et al., 2025).

The investigation carried out on piglet jejunal epithelial cells, IPEC-J2, treated with different concentrations of S-methylmethionine sulfonium chloride has demonstrated that vitamin U could stimulate the expression of Mucin-2 (MUC2), epidermal growth factor (EGF), glucagon-like peptide-2 (GLP-2) and insulin-like growth factor-1 (IGF-1), while inhibit the expression of transforming growth factor beta (TGF-β1) in both mRNA and protein level that may great importance to repair injury and maintain the intestinal mucosa barrier, and promote intestinal development (Yang et al., 2022).

These data suggest that mechanisms other than methylation may also be involved in the gastroprotective effect of vitamin U.

### 3.4 Impact of vitamin U on gastrointestinal disorders

Preclinical and clinical studies using vitamin U underscore its potential therapeutic utility in various gastrointestinal disorders (Cheney, 1950; 1952; Nesterova and Tayts, 1973; Samson and Lukanev, 1973; Ichikawa et al., 2009; Patel and Prajapati, 2012; Kruchinina et al., 2018; Topchiy and Toporkov, 2019; Ivashkin et al., 2020). S-methylmethionine may exert gastroprotective effects through several mechanisms, including enhancement of mucosal blood flow, stimulation of epithelial cell proliferation and growth factors production, increase of mucin secretion, and modulation of gastric acid secretion (Watanabe et al., 1996; Ichikawa et al., 2009; Yang et al., 2022).

Besides these, the anti-inflammatory and antioxidant properties of vitamin U contribute to the preservation of mucosal integrity and attenuation of gastrointestinal injury. At this, the cytoprotective effect of vitamin U may be mediated by sulfhydryl compounds, and the increase in surface mucosal mucin may be related to cytoprotection (Watanabe et al., 1996; Ichikawa et al., 2009; Watanabe et al., 2000; Yang et al., 2022).

The complex research was carried out to investigate the alleviating effects of S-methionine on the intestinal damage induced by heat stress in mice. The results showed that methionine can improve intestinal mucosal morphology (increase the villi height, muscle layer thickness, decrease crypt depth), increase the expression of tight junction proteins (Claudin-1, Occludin, ZO-1) and the content of DAO, decrease the content of intestinal mucosa damage markers (ET, FABP2) and peroxidation products (MDA), increase the activity of antioxidant enzymes (GR, GSH-Px, SOD), the number of goblet cells, the contents of immunoglobulins (sIgA, IgA, IgG, and IgM) and stress protein (HSP70), and the activity of Na+/K+-ATPase. These data suggest that vitamin U can alleviate intestinal damage under heat stress (Feng et al., 2024).

Clinical trials evaluating the efficacy of S-methylmethionine in conditions such as peptic ulcer disease, gastroesophageal reflux disease, and gastritis have demonstrated promising outcomes, suggesting its potential as a complementary therapeutic agent in erosive gastrointestinal disorders (Ivashkin et al., 2020).

The purpose of the other clinical study was to evaluate the effect of S-methylmethionine sulfonium chloride intake on the symptoms of dyspepsia and the quality of life of patients with chronic gastritis. The study included 37 patients (21 men and 16 women) aged 35–60 years with chronic gastritis of various etiologies. All patients were prescribed S-methylmethionine at a dose of 300 mg per day. The study showed that taking a vitamin U dietary supplement for 6 months helps to reduce the severity of dyspeptic symptoms in patients with chronic gastritis and their quality of life (Drozdov et al., 2023).

S-methylmethionine has a beneficial effect also on the function of the liver and gall bladder, and therefore is useful for the treatment of various erosive diseases of these organs (Patel and Prajapati, 2012; Sokmen et al., 2012; Bas et al., 2016; Topaloglu et al., 2022), which also favors its potential in erosive gastrointestinal disorders.

### 3.5 Adverse effects and precautions

S-methylmethionine is rapidly absorbed, accumulated in the liver and kidneys, and actively assimilated by the organisms of rats and man. Vitamin U exists in D- and L-forms. The L-form is used predominantly. The concurrent administration of D- and L-forms of vitamin U stimulates processes of D-form assimilation (Gessler and Bezzubov, 1987).

Vitamin U is safe when eaten directly from whole foods. However, little is known about its safety or potential side effects in diet supplement form. According to the European Chemicals Agency, vitamin U may cause eye, skin, or lung irritation if it comes into direct contact with these organs. Due to limited research, vitamin U dosage recommendations haven’t been established. More research is needed to understand whether an overdose is possible and the signs and symptoms associated with it. There isn’t enough scientific information to determine whether vitamin U interacts with other supplements or drugs (Petre, 2020).

## 4 Justification of combined use of vitamin B5 and vitamin U

**FIGURE 4:**
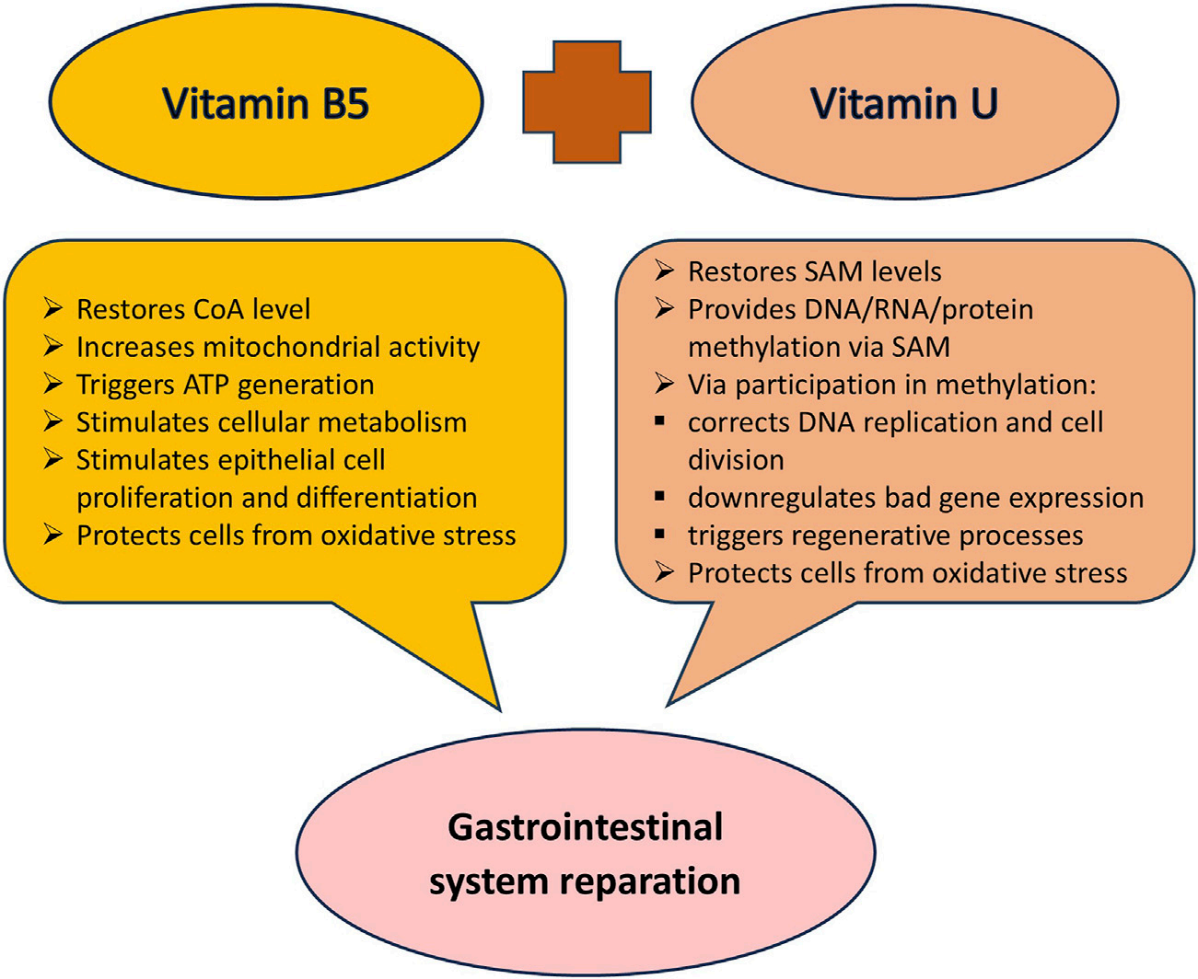
The combined protective impact of vitamin B5 and vitamin U on the gastrointestinal system. Abbreviation: CoA, coenzyme A; SAM, S-adenosyl-L-methionine.

The combined effect of vitamin B5 and vitamin U on the gastroenteric system is mediated through diverse yet complementary mechanisms including mucosal protection, anti-inflammatory actions, and antioxidant and regenerative properties (Figure 4). Synergistic interactions between these two vitamins may potentiate their therapeutic efficacy offering an improved approach to treating inflammatory and erosive gastrointestinal pathologies. The rationality for using this combination proves current knowledge about the participation of vitamin B5 and vitamin U in metabolic and energy processes in cell life and clinical studies.

### 4.1 Scientific arguments

The synergistic use of the anti-ulcer vitamin U (S-methylmethionine) which is an active donor of methyl groups in the cell life cycle and the reparative vitamin B5 (dexpanthenol) participating in the ATP generation allows to simultaneously turn on different biochemical mechanisms for healing the suffering gastrointestinal mucosa. Therefore, this synergistic combination provides the effective performance of many cellular functions necessary for cell and tissue regeneration which include.

**FIGURE 5:**
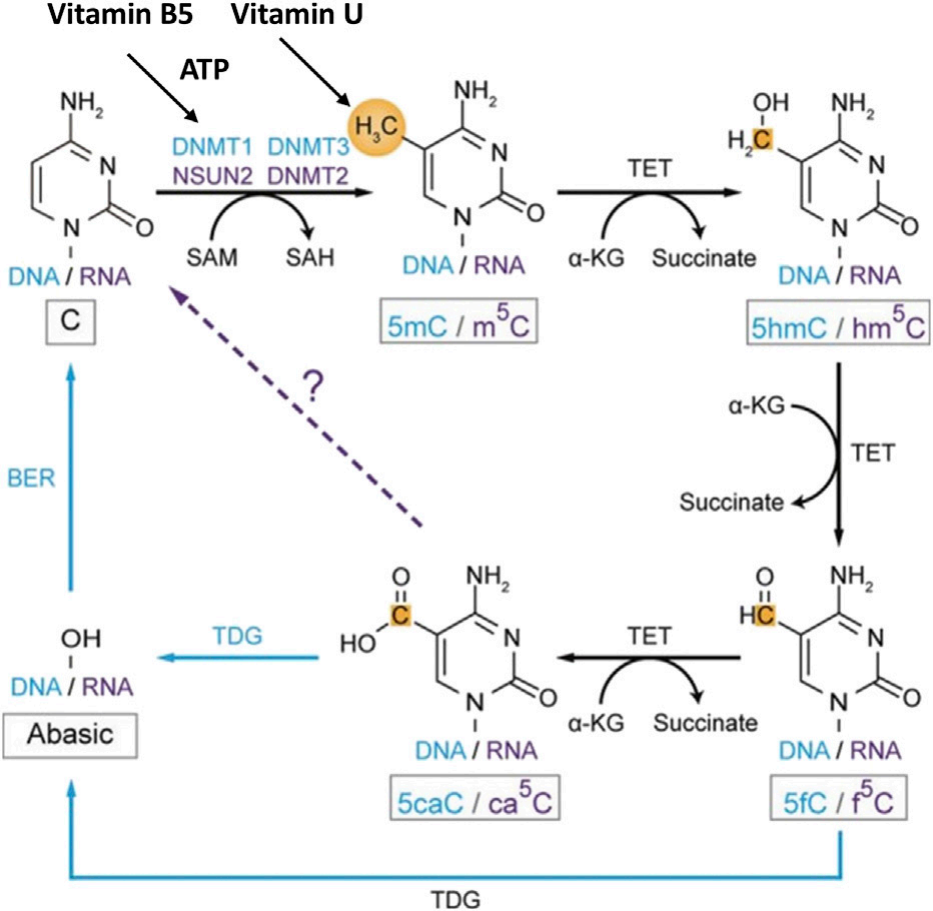
Role of vitamin B5 and vitamin U in DNA and RNA methylation and demethylation in C^5^-cytosine. The blue color shows the DNA methylation pathway. The purple color shows the RNA methylation pathway. The black color shows common components of the methylation pathways. The dashed arrow indicates the potential step. Methyl groups and carbon atoms are shown in gold. Abbreviation: ATP, adenosine 5′-triphosphate; DNMT, DNA methyltransferase; NSUN, NOL1/NOP2/Sun domain RNA methyltransferase family genes; 5mC, C^5^-methylcytosine in DNA; m^5^C, C^5^-cytosine methylation in mRNA; 5caC, C^5^-cytosine carboxylation in DNA; ca^5^C, C^5^-cytosine carboxylation in mRNA; α-KG, α-ketoglutarate; SAM, S-adenosyl-L-methionine; SAH, S-adenosyl-L-homocysteine; TET, ten-eleven translocation subfamily enzymes. Modified from Liu et al. (2023). This open access article is distributed under the terms and conditions of the Creative Commons Attribution (CC BY) license (https://creativecommons.org/licenses/by/4.0/).

1. Conversion of methionine into its active form SAM. This process requires ATP energy provided as a result of vitamin B5 conversation to CoA that donates ADP molecule for the ATP synthesis in the biochemical Krebs cycle (Slyshenkov et al., 2004; Gubergrits et al., 2016; Czumaj et al., 2020; Abdelraheem et al., 2022; Hrubša et al., 2022) (Figure 2).
2. Chromatin decondensation. This process occurs as the methylation result of individual sections of DNA (the donor of CH3 groups is SAM or S-methylmethionine). Chromatin decondensation also requires the participation of ATP energy synthesized in a cycle of pantothenic acid metabolism (Patel and Prajapati, 2012; Abdelraheem et al., 2022; Hrubša et al., 2022; Liu et al., 2023). The role of chromatin decondensation at the start of the regenerative cell cycle is indisputable. It is known that despite the microscopic dimensions of a cell, the total length of DNA molecules packed in a cellular nucleus is up to 2 km. The compact arrangement of DNA is ensured by the formation of complex and dense spatial “tangles” of chromatin. In this case, the DNA packaged in chromatin is inactive and only after decondensation is subject to replication giving rise to the process of cell division (Gubergrits et al., 2016; Lorzadeh et al., 2021) (Figure 3).
3. DNA methylation. The presence of methyl groups in DNA chains is necessary for the formation of chromosome structure and the regulation of gene transcription (Jorgensen and Ro, 2019; Lorzadeh et al., 2021; Abdelraheem et al., 2022; Liu et al., 2023; Wang et al., 2023). When replication is completed the methylation of nucleotide residues of the newly formed DNA chains occurs. A family of DNA methyltransferases (Dnmts) catalyzes the transfer of methyl groups from SAM to the fifth carbon of cytosine residue in the CG sequence resulting in the 5-methylcytosine (5 mC) formation. At this, Dnmt3a and Dnmt3b transfer methyl groups onto naked DNA while Dnmt1 maintains a DNA methylation pattern during replication. While parental DNA undergoes replication, it retains the original methylation. Dnmt1 associates at the replication foci and precisely replicates the original DNA methylation pattern by adding methyl groups onto the newly formed daughter strand (Moore et al., 2013; Wang et al., 2023) (Figure 5). DNA cytosine methylation is the predominant DNA modification and occurs almost exclusively at the symmetric CG dinucleotides in most somatic cells or tissues. Specifically, 60%–80% of CG dinucleotides in the human genome are methylated. The effect of cytosine methylation is context-dependent. Its presence on gene regulatory sequences (promoters or enhancers) usually causes transcriptional silence and may promote transcription when present on gene bodies (Liu et al., 2023) (Figure 3).

DNA methylation has been identified recently also at the nitrogen-6 position of adenosine forming N6-methyladenosine (6 mA). The DNA methylation in adenine in contrast to DNA cytosine methylation is a relatively new epigenetic modification and a low abundance. Studies have reported that 6 mA is implicated in regulation transcription, transposon activity, and other functions (Liu et al., 2023). These DNA modifications use ATP energy provided by pantothenic acid. SAM and possibly S-methionine are used as sources of methyl groups (Patel and Prajapati, 2012; Moore et al., 2013; Gubergrits et al., 2016; Abdelraheem et al., 2022; Hrubša et al., 2022; Liu et al., 2023).

4. RNAs and protein methylation. Beyond epigenetic regulation, methylation of RNAs and proteins provides two additional layers for governing gene expression and function. All types of RNAs including transfer RNA (tRNA), ribosomal RNA (rRNA), messenger RNA (mRNA), micro-RNA (miRNA), and long non-coding RNA (lncRNA) undergo methylation. More than 70 types of RNA methylations on cytosine (methylcytosine, m5C), adenosine (N6 -methyladenosine, m6A), guanosine (N7 -methylguanosine, m7 G) and 2′-O-methyl (Nm) have been identified (Liu et al., 2023) (Figure 5; RNA methylation shown only for cytosine residues). Broad interest in RNA methylation biology has been due to the discovery of the significant level and function of mRNA internal modifications, primarily m6A which is the most abundant one and regulates the mRNA splicing, translation, localization, and stability.

Histone methylation on the side chains of lysine (Lys) and arginine (Arg) residues either upregulates or downregulates transcription depending on the location within histone proteins and the methylation degree. Nonhistone protein methylation also mainly occurs at Lys and Arg residues and shares the common set of enzymes with histone methylation to regulate the subcellular localization, activity, and stability of methylated proteins. Extensive crosstalk among protein, RNA, and DNA methylation in various biological processes generates a sophisticated regulatory network (Liu et al., 2023). Protein molecules are polypeptide chains consisting of individual amino acids. However, free amino acids are not active enough to combine in a polypeptide chain. Therefore, before forming a protein molecule they are activated with the help of special enzymes which are specific for each amino acid (Abdelraheem et al., 2022). The energy source for this is ATP. As a result of activation, the amino acid becomes more labile and, under the action of the same enzyme, binds to tRNA for further stages of cellular metabolism. Each of the 20 amino acids of the protein is connected by covalent bonds to a specific tRNA also using the energy of ATP. SAM in tern participates in all biosynthetic reactions using the methyl groups. The S-adenosylhomocysteine formed after the cleavage of the methyl group undergoes hydrolysis into adenosine and homocysteine; the latter is used to synthesize serine. This is the main conversion route (Patel and Prajapati, 2012; Gubergrits et al., 2016; Abdelraheem et al., 2022; Hrubša et al., 2022; Wang et al., 2023). Thus, both vitamins are essential in DNA, RNA, and protein synthesis as the main structure and functional cellular compounds.

### 4.2 Clinical studies

The rationality for using the combination of vitamin B5 and vitamin U and its therapeutic effectiveness for the treatment of post-eradication chronic erosive and ulcerative disease of stomach and duodenum associated and non-associated with *H. pylori* infection is supported by clinical studies and presented in publications leading Ukrainian gastroenterologists (Gubergrits et al., 2013; 2014; 2016; Danylchenko and Tokarenko, 2014; Bondarenko et al., 2015; Khristich, 2016; Kolesnikova and Solomentseva, 2017; Babinets et al., 2019; Babinets and Machnitska, 2019; 2021). These clinical investigations also underscore the safety and well-tolerability profile of the vitamin B5 and vitamin U combination supporting the expediency of its inclusion as an adjunctive therapy in clinical practice for erosive gastrointestinal diseases. In particular, the diet supplement Doktovit (a combination of 100 mg of vitamin U and 50 mg of vitamin B5) is recommended for use in the complex treatment of acid-dependent diseases of the gastrointestinal tract (chronic pancreatitis, chronic erosive gastritis, gastroesophageal reflux disease associated and non-associated with *H. pylori* infection). As believed, this combination provides a cytoprotective and regenerative effect on the mucous membrane of the stomach, and duodenum, especially in erosive and ulcerative lesions, and normalizes pancreas function (Danylchenko and Tokarenko, 2014; Kolesnikova and Solomentseva, 2017; Babinets et al., 2019; Babinets and Machnitska, 2021). With this aim, the improved analogous of Doktovit registered by a Cyprus company, IK Eurika Ltd., under the trademark Gastro-Eurika can be also used.

In the study of Gubergrits and coauthors (Gubergrits et al., 2013), forty patients with erosive and ulcerative diseases of the stomach and duodenum infected with *H. pylori* were examined. Patients were observed twice - before treatment and 4 weeks after the end of anti-Helicobacter therapy. The patients were divided into two treatment groups of 20 patients each. The basis of treatment was the classic triple anti-Helicobacter therapy regimen consisting of rabeprazole (20 mg) 2 times a day, clarithromycin (500 mg) 2 times a day, and amoxicillin (1,000 mg) 2 times a day for 10 days. In addition to anti-Helicobacter therapy, patients of the main group received Doktovit in a dose of 1 tablet 3 times a day after meals for 1 month. Already during the first 3 days of therapy, the advantages of treatment with the inclusion of Doktovit concerning the relief of pain syndrome were revealed. Thus, in patients of this group, the pain disappeared in 70% of cases, decreased in 20%, and remained the same only in 10% *versus* patients of the comparison group taking the regular therapy respectively, in 60%, 20%, and 20% of cases. Disappearance or significant reduction of clinical manifestations of gastroduodenal pathology at the end of treatment in the main group occurred in 95% (19 patients), and in the comparison group in 85% (17 patients). The frequency of *H. pylori* eradication in the main group was 90% (18 patients) and 85% (17 patients) in the comparison group. Eradication of *H. pylori* was not achieved in 2 (10%) and 3 (15%) patients, respectively, which was not statistically significant. Four weeks after the treatment ended, the frequency of erosion epithelialization and ulcer scarring was 95% (19 patients) in the main group and 80% (16 patients) in the comparison group. According to the authors of this study, the inclusion of Doktovit in the basic treatment regimen helps to reduce the severity of clinical manifestations of erosive and ulcerative lesions of the gastroduodenal zone, increase the frequency of their healing and the tendency to improve the psychosomatic status of patients (Gubergrits et al., 2013).

In the other study by Gubergrits and coauthors (Gubergrits et al., 2014), thirty-two patients were observed to evaluate the dynamics of histological changes in the gastric mucosa in patients with erosive and ulcerative changes in the gastroduodenal zone under the influence of treatment with the inclusion of Doktovit. *Helicobacter pylori* infection was diagnosed in 25 (78.1%) patients. In 7 (21.9%) patients there was an indication of taking nonsteroidal anti-inflammatory drugs. All patients received proton pump inhibitors in a standard dose and eradication therapy in cases of *H. pylori* detection. In addition, all patients were prescribed Doktovit in a dose of 1 tablet daily for a month. The comparison group included 28 patients with similar pathology who received the same treatment but without the inclusion of Doktovit. Biopsies of the gastric mucosa of the antral and pyloric sections of the stomach were evaluated. To diagnose *H. pylori* infection, the number of bacteria in the mucus and the stomach epithelium was assessed. The authors noted an improvement in the morphological state of the gastric mucosa after treatment with Doktovit stating that lymphoid follicles were not found in the mucosa and a decrease in the severity of inflammatory cellular infiltration of the lamina propria of the mucosa was detected in all patients (Gubergrits et al., 2014).

In the study of Danylchenko and Tokarenko (Danylchenko and Tokarenko, 2014), sixty patients who were hospitalized with the confirmed diagnosis of gastroesophageal reflux disease accompanied by *Helicobacter* pylori-associated erosive gastroduodenitis were observed to present the grounds for the inclusion of Doktovit in the therapeutic complex at erosive and ulcerative digestive, gastric, and duodenal injuries against the background of standard therapy. The patients of the main group (30 patients) received Doktovit in a dose of 1 tablet three times a day and standard triple therapy (proton pump inhibitors, clarithromycin, amoxicillin) and suspension of sodium alginate. The patients of the comparison group (30 patients) received standard triple therapy and suspension of sodium alginate without Doktovit. The follow-up period was 1.5 months. All patients underwent biopsy with subsequent histological investigations for *H. pylori* exposure. After 12 days from the therapy start, the complete epithelization of erosive defects has been revealed in 90% of patients in the main group. In other patients of this group, the control fiberoptic esophagogastroduodenoscopy revealed the apparent dynamics of the erosion dimensions reduction (2.3 times). At the same time, in the comparison group, the complete epithelization of erosive defects was gained only in 60% of patients, and the rest showed a significant erosion dimensions reduction (1.6 times). The results of esophagogastroduodenoscopy and histological investigations after 12 days of treatment showed the removal of inflammatory mucosa changes in 85% of patients of the main group and 55% of the comparison group. In the authors’ conclusion, Doktovit considerably increased the efficacy of the standard basic therapy and stimulated the regenerative processes, thus promoting the faster healing of erosions and mucosa epithelization, removal of the mucosa inflammation in the esophagus and stomach, relief of clinical symptoms of the disease and reduction of the treatment duration (Danylchenko and Tokarenko, 2014).

In the study of Bondarenko and coauthors (Bondarenko et al., 2015), forty-eight patients with gastroesophageal reflux disease were observed. Patients were evenly divided into two groups. The main group of 24 patients received treatment with pantoprazole 40 mg 2 times a day and Doktovit in a dose of 1 tablet 3 times a day. A comparison group of 24 patients received pantoprazole monotherapy at a dose of 40 mg 2 times a day. The duration of both treatment regimens was 8 weeks. It was established that against the background of treatment of the erosive form of gastroesophageal reflux disease with the use of a combination of pantoprazole and Doktovit, there is a faster regression of the intensity of clinical signs of gastroesophageal reflux disease, especially heartburn and accompanying dyspeptic manifestations of the disease, 87.5% (21 patients) *versus* 79.0% (19 patients) in the control group (Bondarenko et al., 2015).

In the study of Kolesnikova and Solomentseva (Kolesnikova and Solomentseva, 2017), sixty patients with gastropathy caused by taking nonsteroidal anti-inflammatory drugs (no-associated with *H. pylori*) were observed to investigate the effect of Doktovit on the clinical symptoms, frequency of epithelization ulcers, and erosions, as well as the dynamics of morphological changes of the gastric mucosa in patients. Patients of the main group (30 patients) received a proton pump inhibitor pantoprazole (40 mg) 2 times a day and Doktovit in a dose of 1 tablet 3 times a day. Patients of the comparison group (30 patients) received only pantoprazole in a standard daily dose for 30 days. All patients underwent a clinical examination, fibrogastroduodenoscopy of the esophagus, stomach, and duodenum, and histological examination of the gastric mucosa biopsies. A faster normalization of clinical symptoms was noted in patients who received Doktovit with pantoprazole. By the end of the third week of treatment, there was a statistically significant lower number of patients with complaints. The treatment regime with Doktovit contributed to the inflammatory process decreasing in the mucous membrane of the stomach and duodenum, and the epithelization of erosions. Finally, the erosive damages were not observed in 29 (96%) patients of the main group while in the comparison group only 22 (73%) patients had the same effect. Doktovit was well tolerated and did not cause side effects (Kolesnikova and Solomentseva, 2017).

In the studies of Babinets and coauthors (Babinets et al., 2019), Babinets and Makhnitska (Babinets and Machnitska, 2021), forty-five patients with chronic pancreatitis and *Helicobacter* pylori-associated chronic gastritis were observed. The main group included 25 patients and the control group was 20 patients. All patients received 10-day therapy with pantoprazole in a dose of 40 mg 2 times a day, amoxicillin in a dose of 1,000 mg (or metronidazole in a dose of 500 mg) 2 times a day, clarithromycin in a dose of 500 mg 2 times a day. Patients of the main group received additional vitamin complex Doctovit in a dose of 2 tablets a day after meals for 2 months. The esophagogastroduodenoscopy, urease test for *H. pylori*, and biopsy from 5 sites with histological examination were performed at the beginning and 2 months after the treatment. It was found a significant decrease in lymphohistiocytic infiltration in the gastric mucosa, restoration of the structure of the pancreas, and increased focal proliferation of the glandular epithelium which were considered as the signs of morphological restoration of the epithelium and reduction of the signs of its dysplasia. There was a proven higher effectiveness of the pancreas exocrine insufficiency correction when the treatment with Doktovit (28.12%) *versus* without Doktovit (20.74%) was used. The total dynamics of improvement of the morphological conditions of the gastric mucosa in the group with Doktovit was 32% *versus* 17% in the group without Doctovit (Babinets et al., 2019; Babinets and Machnitska, 2021).

## 5 Prospects and conclusions

The studies evaluating vitamin B5 and vitamin U as potential therapeutic agents in inflammatory and erosive gastrointestinal pathologies represent a promising avenue for advancing patient care. Future research endeavors should focus on detailing the underlying mechanisms of their action, conducting large-scale clinical trials to validate the efficacy of this combination, and refining therapeutic strategies to maximize clinical outcomes. Further research is needed to elucidate optimal dosing regimens, long-term safety profiles, and potential drug interactions to facilitate their integration into standardized treatment protocols. Additional preclinical investigation using humanized animal models and *in vitro* models of cell cultures, organoids, and organs-on-chips comparing the benefits of the combined action of vitamin B5 and vitamin U *versus* their effects alone might greatly impact the justification of combined therapy.

Moreover, efforts to enhance public awareness and healthcare provider education regarding the role of micronutrients in gastrointestinal health are also highly relevant. By harnessing the therapeutic potential of vitamin B5 and vitamin U, we can strive towards optimizing the management of inflammatory and erosive gastrointestinal diseases and improving patient outcomes.

The combination of vitamin B5 and vitamin U presented in the drug Doktovit and its improved analogous Gastro-Eurika (IK Eurika Ltd., Limassol, Cyprus) holds promise as an effective adjunctive therapy for erosive gastroduodenitis and other gastrointestinal pathologies associated with mucous lesions. This combination offers multifactor reparative and gastroprotective effects through diverse and synergic mechanisms of action. While further research has to be engaged to validate the benefits and clinical efficacy of using the combined drag of vitamin B5 and vitamin U and elucidate optimal therapeutic strategies, presented in this work scientific data, insights, and clinical evidence highlight the undoubted benefits of including this vitamin complex in the standard scheme of erosive gastrointestinal disease treatment as associated and non-associated with *H. pylori* infection.
